# Tumor Vasculature-Targeted Recombinant Mutated Human TNF-α Enhanced the Antitumor Activity of Doxorubicin by Increasing Tumor Vessel Permeability in Mouse Xenograft Models

**DOI:** 10.1371/journal.pone.0087036

**Published:** 2014-01-22

**Authors:** Changli Jiang, Junzhou Niu, Meng Li, Yi Teng, Huixuan Wang, Yingqi Zhang

**Affiliations:** 1 Clinical Laboratory, Army Center for Molecular Biological Analysis, Kunming General Hospital of Chengdu Military Area, Kunming, Yunnna, China; 2 The State Key Laboratory of Cancer Biology, Department of Biopharmaceutics, School of Pharmacy, Fourth Military Medical University, Xi’an, Shaanxi, China; 3 Department of Dermatology, Xijing Hospital, Fourth Military Medical University, Xi’an, Shaanxi, China; Virginia Commonwealth University Medical Center, United States of America

## Abstract

**Objective:**

Increasing evidence suggests that, when used in combination, tumor necrosis factor-α (TNF-α) synergizes with traditional chemotherapeutic drugs to exert a heightened antitumor effect. The present study investigated the antitumor efficacy of recombinant mutated human TNF-α specifically targeted to the tumor vasculature (RGD-rmhTNF-α) combined with the chemotherapeutic agent doxorubicin in 2 murine allografted tumor models.

**Methods:**

Mice bearing hepatoma or sarcoma allografted tumors were treated with various doses of RGD-rmhTNF-α alone or in combination with doxorubicin (2 mg/kg). We then evaluated tumor growth and tumor vessel permeability as well as intratumoral levels of RGD-rmhTNF-α and doxorubicin.

**Results:**

RGD-rmhTNF-α treatment enhanced the permeability of the tumor vessels and increased intratumoral doxorubicin levels. In addition, intratumoral RGD-rmhTNF-α levels were significantly higher than that of rmhTNF-α. In both of the tested tumor models, administering RGD-rmhTNF-α in combination with doxorubicin resulted in an enhanced antitumor response compared to either treatment alone. Double-agent combination treatment of doxorubicin with 50,000 IU/kg RGD-rmhTNF-α induced stronger antitumor effects on H22 allografted tumor-bearing mice than the single doxorubicin agent alone. Moreover, doxorubicin with 10,000 IU/kg RGD-rmhTNF-α synergized to inhibit tumor growth in S180 allografted tumor-bearing mice.

**Conclusions:**

These results suggest that targeted delivery of low doses of RGD-rmhTNF-α into the tumor vasculature increases the antitumor efficacy of chemotherapeutic drugs.

## Introduction

Tumor necrosis factor-α (TNF-α) exhibits potent antitumor activity, alters endothelial barrier function, reduces tumor interstitial pressure, and mediates immune responses [1]. However, systemic TNF-α administration for antitumor therapy is accompanied by prohibitive toxicity, where the maximum tolerated dose (8–10 µg/kg) is 10 to 50 times lower than the estimated effective dose [2]–[4]. For this reason, systemic TNF-α administration has been abandoned as a viable therapy, and its clinical use has been limited to locoregional treatments [5], [6]. To overcome this limitation of toxicity, an effort has been made to create a TNF mutant by protein-engineering methods that retains the antitumor ability of TNF-α but exhibits decreased toxicity [7]–[9]. One such TNF-α mutant, the recombinant mutated human TNF-α (rmhTNF-α), was generated by deleting the first seven amino acids at the N-terminus and replacing the Pro 8, Ser 9, and Asp 10 with Arg 8, Lys 9, and Arg 10, respectively, as well as Leu 157 with Phe 157 at the C-terminus. We previously reported that rmhTNF-α treatment increased antitumor activity with reduced toxicity in H22 hepatoma and S180 sarcoma allografted mice [10].

In solid tumors, the progressive growth and metastasis of malignant neoplasms depend upon the formation of new blood vessels. Tumor vasculature differs both functionally and morphologically from the vasculature in normal tissues, where tumor blood vessels are generally more heterogeneous in distribution, larger in size, and more permeable [11]. Drug delivery, transport, and spatial distribution in solid tumors are affected by multiple physicochemical and biologic factors, some of which are dynamic properties that change with time and drug treatment. A better understanding of the contributions of these various factors might lead to therapeutic strategies that permit passive and/or active tumor targeting [11]. For example, chemotherapeutic agents must enter the tumor blood vessels, cross the vessel wall, and finally migrate through the interstitium to reach cancer cells in solid tumors.

Integrin αvβ3 is an attachment molecule that is usually expressed at low levels on epithelial and mature endothelial cells [12] but is overexpressed on activated endothelial cells in the neovasculature of numerous carcinomas, including hepatocellular carcinoma and sarcoma [13]–[16]. The tumor-homing peptide RGD-4C (CDCRGDCFC) selectively binds to αvβ3 and αvβ5 integrins, making it able to home to several different tumor types in a highly selective manner [17]. Because of this property, RGD-based strategies have been used extensively as a way to selectively deliver therapeutics and imaging agents to tumors [18]. Indeed, coupling anticancer drugs or peptides to RGD peptides yields compounds that exhibit increased antitumor activity with lowered toxicity to normal tissues in mice [19].

In a previous study, we generated the RGD-rmhTNF-α molecule, confirmed that it could bind to αvβ3 integrin *in vitro,* and found that its bioactivity was similar to that of rmhTNF-α [20]. With the aim of further improving the potential clinical application of RGD-rmhTNF-α, we hypothesized that coupling the RGD-4C peptide to rmhTNF-α would selectively target it to tumor vessels *in vivo*, increase tumor vessel permeability, and promote the antitumor activity of chemotherapeutic agents within the tumor microenvironment. In the present study, we addressed our hypothesis in the murine H22 hepatoma and S180 sarcoma allografted tumor models. We found that intratumoral levels of RGD-rmhTNF-α were significantly higher than that of rmhTNF-α. Furthermore, RGD-rmhTNF-α enhanced tumor vessel permeability. When combined with the traditional chemotherapeutic drug doxorubicin, RGD-rmhTNF-α increased intratumoral doxorubicin levels and synergized with doxorubicin to enhance the antitumor activity of this chemotherapy. These results suggested that targeted delivery of low doses of RGD-rmhTNF-α to the tumor vasculature increased the therapeutic efficacy of chemotherapeutic drugs for solid tumors.

## Materials and Methods

### Drugs and Reagents

rmhTNF-α and RGD-rmhTNF-α were prepared by recombinant DNA technology and were purified from *Escherichia coli* cell extracts as previously described [20], [21]. rmhTNF-α and RGD-rmhTNF-α were purified to 97.5% and 95% purity, respectively, by high-performance liquid chromatography. Various concentrations of purified rmhTNF-α, RGD-rmhTNF-α, and doxorubicin hydrochloride were diluted with normal saline. rmhTNF-α bioactivity was estimated as 0.1–1.2×10^9^ IU/mg using standard procedures on the mouse fibroblast cell line L929. RGD-rmhTNF-α bioactivity was estimated as 0.1–0.8×10^9^ IU/mg. Doxorubicin hydrochloride for injection (10 mg/unit, stored at room temperature) was purchased from Shenzhen Main Luck Pharmaceuticals Incorporation (China). Evans Blue due was supplied by Fluka Incorporation (Switzerland). Murine anti-hTNF-α monoclonal antibodies were kindly provided by the Immunology Department at the Fourth Military Medical University (Xi’an, China).

### Cell Lines

The H22 murine hepatoma cell line (Department of Biopharmaceutics, School of Pharmacy, The Fourth Military Medical University, China) was kept in liquid nitrogen and passaged in the abdominal cavity of the BALB/c mice. The S180 murine sarcoma cell line (Department of Biopharmaceutics, School of Pharmacy, The Fourth Military Medical University, China) was kept in liquid nitrogen and passaged in the abdominal cavity of Kunning mice. BALB/c and Kunning mice were purchased from the Experimental Animal Center of Lanzhou Medical University (Lanzhou, China). Thawed cells were maintained in RPMI 1640 (Gibco/BRL Invitrogen) supplemented with 2 mM L-glutamine, 10% heat-inactivated fetal calf serum (FCS, purchased from Hyclone) and penicillin/streptomycin (100 U/mL and 100 µg/mL, respectively) in a humidified atmosphere of 5% CO_2_ at 37°C. Three days later, 0.08 mL (1×10^8^ cells/mL) of H22 cells in RPMI 1640 was inoculated into the abdominal cavity of BALB/c mice, and S180 cells were inoculated into the abdominal cavity of Kunming mice for passage maintenance. Eight days after inoculation, ascites cells were collected using a syringe and 7-gauge needle under aseptic conditions, and a tumor cell suspension (2.5×10^6^ cells/mL) was prepared in normal saline (NS).

### Tumor Allograft Models

Male and female ICR mice (8–12-weeks-old; 18–22 g) were obtained from the Mataria Medical Institute (Chinese Academy of Medicine, Beijing, China; Certificate No. Beijing 01–3007) and used as the tumor-bearing mice. Mice were challenged with 0.2 mL of live mouse H22 hepatoma or S180 sarcoma cells from ascites (harvested 8 days after inoculation, as above) by subcutaneous (s.c.) injection into the left flank.

Mice were maintained in an air-conditioned barrier facility at an ambient temperature of 25±2°C, a relative humidity of 50±10%, and a 12-h on/off light cycle. Health was monitored daily by gross observation. Mice were treated humanely, and all study protocols were performed in accordance with the Regulations of Good Laboratory Practice for nonclinical laboratory studies of drugs issued by the National Scientific and Technologic Committee of People’s Republic of China. The study protocol was approved by Lanzhou Medical University Ethics Committee. All surgery was performed under sodium pentobarbital anesthesia, and all efforts were made to minimize suffering.

### Detection of Evans Blue and Doxorubicin in Tumors

Vessel permeability was assessed using an Evans blue dye assay. S180-bearing ICR mice (tumor diameter, 1.0–1.5 cm) were treated intramuscularly (i.m.) with RGD-rmhTNF-α (0.1 mL), followed 2 h later by administration of either intravenous (i.v.) Evans Blue (0.1%, 0.2 mL) or intraperitoneal (i.p.) doxorubicin (2 mg/kg); mice receiving Evans Blue dye or doxorubicin alone were used as the respective controls. After 2 h, mice were sacrificed, and tumors were excised and divided into 3 sections of approximately equal size. Each tumor section was weighed, homogenized, resuspended in cold PBS containing 1% Triton X-100 (1 mL/g tumor), and incubated for 1 h on ice. The suspension was then centrifuged (14,000×*g*, 4°C, 15 min), and the supernatants were separated for subsequent assays. For Evans Blue detection, supernatant was mixed with acetone (10% [v/v] final concentration). The product was centrifuged again (14,000×*g*, 4°C, 15 min), and absorbance was measured at 620 nm using a 960MC spectrophotometer (Shanghai, China). The relative increase in Evans Blue dye was calculated as compared to administration of Evans Blue alone, as follows: relative increase (%) = [concentration of Evans blue dye_(RGD-rmhTNF-α+Evans Blue group)_- concentration of Evans blue dye_(Evans Blue group)_]/concentration of Evans blue dye_(Evans Blue group)._

To measure intratumoral doxorubicin concentration, the silver nitrate method was used [22]. Supernatant was used to measure maximal fluorescence intensity (Fmax) (doxorubicin itself has a natural fluorescence). Briefly, 0.2 mL of AgNO_3_ (w/v 33%) was added to 1 mL of doxorubicin in aqueous solution or 20% tumor-tissue homogenate and vortexed for 10 min. Pre-cooled isoamyl alcohol (4 mL) was added to the homogenate, and the mixture was centrifuged for 10 min (5000×*g*). Samples were analyzed in duplicate and were compared against the standards. The concentration of Evans blue dye or doxorubicin within the tumor (µg/g tumor tissue) was calculated by averaging the extraction values for each section of tumor. The relative increase in doxorubicin was calculated as compared to administration of doxorubicin alone, as follows: relative increase (%) = [concentration of doxorubicin_(RGD-rmhTNF-α+doxorubicin group)-_ concentration of doxorubicin_(doxorubicin group)_]/concentration of doxorubicin_(doxorubicin group)._

### In vivo Antitumor Activity

ICR mice were challenged by s.c. injection of 5×10^5^ H22 or S180 cells into the left flank. Ten days later, tumor-bearing mice were randomly divided into different groups (n = 8/group) and treated based on the different experimental treatment groups. These treatment groups included: saline control, rmhTNF-α alone, RGD-rmhTNF-α alone, rmhTNF-α plus doxorubicin, and RGD-rmhTNF-α plus doxorubicin. Doxorubicin was administered by i.p. injection at a dose of 2 mg/kg of body weight 2 h after rmhTNF-α or RGD-rmhTNF-α, and mice were treated once every 2 days for a total of 6 consecutive treatments [23], [24]. H22 allografted mice received 5,000, 50,000, or 500,000 IU/kg of rmhTNF-α or RGD-rmhTNF-α, and S180 allografted mice received 10,000, 50,000, or 250,000 IU/kg by the i.p. or i.m. routes of administration. Tumor growth was monitored on a daily basis by measuring tumor volumes with calipers. Tumor volume was calculated using the following formula: ½×length×width^2^. Animals were sacrificed before tumors in the saline groups reached 2.0 to 3.0 cm^3^. Tumors were excised and immediately weighed. Antitumor activity was evaluated by calculating changes in tumor weight. The inhibition rate (IR) was determined using the following formula: IR = (1-T/C)×100(%), where T is the mean tumor weight of the treatment group and C is the mean tumor weight of the control group. All experiments were repeated 3 times.

### Determination of Intratumoral and Serum RGD-rmhTNF-α and rmhTNF-α Levels by Enzyme-linked Immunosorbent Assay (ELISA)

Mice bearing S180 sarcoma allografts were i.v. injected through the tail vein with equimolar amounts of RGD-rmhTNF-α (5.89 µg/kg weight) and rmhTNF-α (5.52 µg/kg weight). At 5, 20, and 60 min post-treatment, mice (n = 6/time point) were sacrificed, blood was harvested from the orbital venous plexus, and tumor tissues were collected. Serum was harvested from the blood. Tumor tissues (100 mg) were homogenized at 2°C to 8°C in order to yield the homogenate. Actual tumor weight was recorded if the tumor weight did not reach 100 mg. RGD-rmhTNF-α and rmhTNF-α levels were determined using the Human TNF-α ELISA Kit (Catalogue Number: EL10019, ANOGEN, Canada). Two TNF-α mouse monoclonal antibodies against different epitopes were used as capture and detecting antibodies. The horseradish peroxidase-labeled anti-mouse IgG was supplied by the kit. The optical density (OD) was read within 30 minutes at 450 nm using an EL*_X_*800 microtiter plate reader (BIO-TEC, USA), and RGD-rmhTNF-α and rmhTNF-α levels were determined according to sample absorbance and the standard curve.

### Statistical Analysis

The Student’s *t*-test was used to determine the significance between each experimental group and was assessed by the DAS software developed by the Chinese Society of Pharmacology (Beijing, China). The results were expressed as the mean ± standard deviation (SD), and the differences between the groups were considered statistically significant when a *p* value of less than 0.05 was achieved.

## Results

### RGD-rmhTNF-α Increased Intratumoral Vascular Permeability and Penetration of Doxorubicin in a Murine Sarcoma Tumor Model

**Figure 1:**
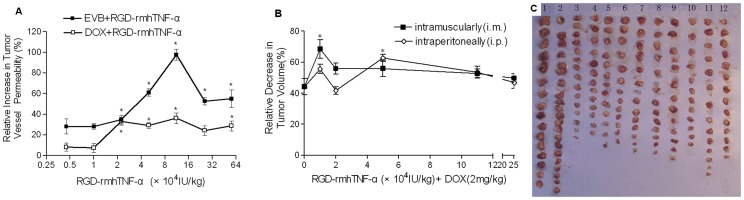
Effect of RGD-rmhTNF-α on tumor vessel permeability, and the effect of doxorubicin combined with RGD-rmhTNF-α on the tumor-growth inhibition rate when administered via different routes. (A) RGD-rmhTNF-α increases tumor-vessel permeability. Mice-bearing S180 cells (n = 8/group) were treated with or without RGD-rmhTNF-α (0.1 mL, i.m.), followed 2 h later by administration of Evans Blue dye (0.1%, 0.2 mL, i.v.) or doxorubicin (2 mg/kg, i.p.). After 2 h, mice were sacrificed, tumors were excised, and the concentration of Evans blue or doxorubicin was measured within the tumor (µg/g tumor tissue). The relative increase in concentration was calculated and statistically analyzed (*p<0.05, RGD-rmhTNF-α+Evans Blue or RGD-rmhTNF-α+doxorubicin compared with Evans Blue or doxorubicin alone, respectively). (B) The effect of RGD-rmhTNF-α combined with doxorubicin on tumor-growth inhibition rate was similar between the i.m. and i.p. administration routes of RGD-rmhTNF-α. Animals bearing S180 allografted tumors (n = 16/group,n = 24 in DOX alone group) were i.m. or i.p. treated every 2 days with various doses of RGD-rmhTNF-α combined with doxorubicin starting on day 10 after tumor implantation. Saline was used as negative control. Mice received a total of 6 consecutive treatments and were sacrificed; tumors were immediately excised and weighed. The inhibition rate was calculated based on the tumor growth of mice in the saline-treated group. Data are represented as the mean (%) ± SD from 3 experiments. (C) Picture showing tumors excised from mice used to calculate the data shown in B. 1, Saline; 2, DOX alone (2 mg/kg); 3–7, DOX+RGD-rmhTNFα (10,000, 22,360, 50,000, 111,800, and 250,000 IU/kg, respectively), i.m.; 8–12, DOX+RGD-rmhTNFα (10,000, 22,360, 50,000, 111,800, and 250,000 IU/kg, respectively), i.p.

To determine whether attaching the tumor-vessel–homing molecule RGD to rmhTNF-α could direct it to tumor vessels and increase tumor neovessel permeability, we injected Evans Blue dye (i.v.) into S180-bearing mice with and without RGD-rmhTNF-α (i.m.) and measured Evans Blue levels in S180 allografted tumors 2 h later. Indeed, co-administration of RGD-rmhTNF-α enhanced the intratumoral level of Evans Blue in a dose-dependent manner, suggesting that RGD-rmhTNF-α increased vascular permeability (Figure 1A). We then tested whether RGD-rmhTNF-α could increase the entry of a chemotherapeutic agent into the tumor and found that co-administration of RGD-rmhTNF-α with doxorubicin increased the intratumoral levels of doxorubicin compared to doxorubicin treatment alone. However, the dose-response curves resembled a bell-shaped curve, indicating that the optimal dose for inducing vascular permeability was in the mid-range of the tested RGD-rmhTNF-α doses. For instance, the intratumoral Evans Blue concentration significantly increased over Evans Blue alone between 22,360 to 559,600 IU/kg of RGD-rmhTNF-α, whereas the intratumoral doxorubicin concentration significantly increased over doxorubicin alone only in the range of 22,360 to 111,806 IU/kg. Thus, the RGD-rmhTNF-α–mediated effect on tumor vascular permeability reached its maximum at a dose of 111,806 IU/kg weight in this experiment. These results suggested that RGD-rmhTNF-α enhanced the permeability of tumor blood vessels and increased the penetration of a chemotherapeutic drug into the tumors.

### Combination Treatment of Doxorubicin with Low Doses of RGD-rmhTNF-α Inhibited Tumor Growth via Various Administration Routes

Although various administration routes and delivery systems have been used in previous studies to deliver TNF-α, few have focused on reporting the most efficient delivery strategies. To determine the most effective route to deliver RGD-rmhTNF-α for improved antitumor function of doxorubicin in our tumor model, we compared the antitumor activity between i.m. and i.p. administration for RGD-rmhTNF-α. Mice receiving combined treatment exhibited significantly higher inhibition of tumor growth than those in the saline-treated control group via both the i.m. and i.p. routes of administration. However, combined treatment exhibited significantly increased inhibition of tumor growth over doxorubicin treatment alone only at the 10,000 IU/kg dose for the i.m. route and 50,000 IU/kg for the i.p. route (*p*<0.05, Figure 1B). No significant difference in the tumor inhibition rate was observed between the i.m. and i.p. routes (*p*>0.05), indicating that the antitumor effect of RGD-rmhTNF-α was similar independent of whether it was administered by the i.m. or i.p. route. These results could also be observed by looking at the gross tumor growth in the representative images from the experimental groups shown in Figure 1C.

### Dose-response Curves of Antitumor Activity by rmhTNF-α and RGD-rmhTNF-α Alone in Murine Hepatoma and Sarcoma Models

**Figure 2:**
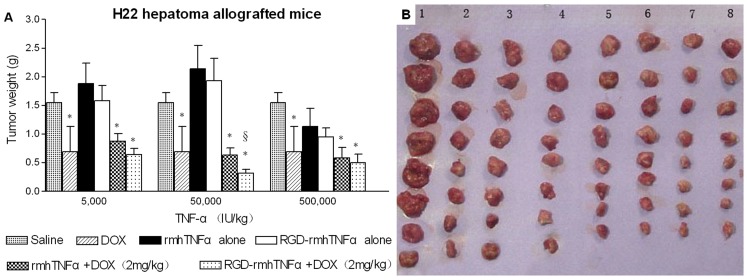
Effect of rmhTNF-α or RGD-rmhTNF-α alone or in combination with doxorubicin on tumor growth in H22 hepatoma-bearing mice. (A) Low doses of RGD-rmhTNF-α most significantly enhanced the therapeutic effects of doxorubicin on allografted H22 hepatomas in mice. Animals bearing tumors (n = 8/group) were i.m. treated every 2 days with various doses of rmhTNF-α alone, RGD-rmhTNF-α alone, rmhTNF-α combined with doxorubicin, or RGD-rmhTNF-α combined with doxorubicin starting on day 10 after tumor implantation. Saline and doxorubicin (doxorubicin, 2 mg/kg weight, i.p.) were used as negative and positive controls, respectively. Mice received a total of 6 consecutive treatments and were sacrificed before tumors in the saline control group reached 2.0 to 3.0 cm^3^; tumors were immediately excised and weighed. Tumor weights are shown as the mean ± SD. (DOX: doxorubicin; *: vs. the saline group; §: vs. the DOX group). (B) Picture showing tumors excised from selected groups used to calculate the data shown in A. 1, Saline; 2, DOX alone (2 mg/kg); 3–5, DOX+rmhTNFα (5,000, 50,000, and 500,000 IU/kg, respectively); 6–8, DOX+RGD-rmhTNFα (5,000, 50,000, and 500,000 IU/kg, respectively).

**Figure 3:**
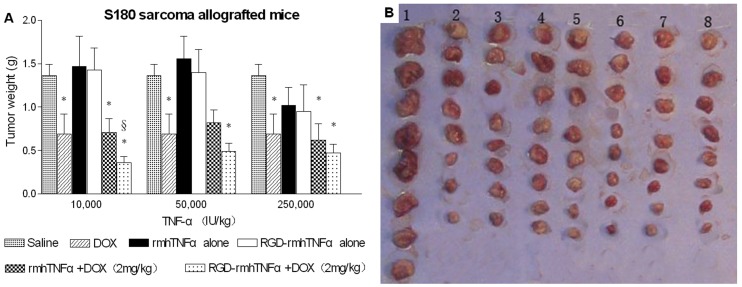
Effect of rmhTNF-α or RGD-rmhTNF-α alone or in combination with doxorubicin on tumor growth in S180 sarcoma-bearing mice. (A) Low doses of RGD-rmhTNF-α most significantly enhanced the therapeutic effects of doxorubicin on allografted S180 sarcomas in mice. Animals bearing tumors (n = 8/group) were i.m. treated every 2 days with various doses of rmhTNF-α alone, RGD-rmhTNF-α alone, rmhTNF-α combined with doxorubicin, or RGD-rmhTNF-α combined with doxorubicin starting on day 10 after tumor implantation. Saline and doxorubicin (doxorubicin, 2 mg/kg weight, i.p.) were used as negative and positive controls, respectively. Mice received a total of 6 consecutive treatments and were sacrificed before tumors in the saline control group reached 2.0 to 3.0 cm^3^; tumors were immediately excised and weighed. Tumor weights are shown as the mean ± SD. (DOX: doxorubicin; *: vs. the saline group; §: vs. the DOX group). (B) Picture showing tumors excised from selected groups used to calculate the data shown in A. 1, Saline; 2, DOX alone (2 mg/kg); 3–5, DOX+rmhTNFα (10,000, 50,000, and 250,000 IU/kg, respectively); 6–8, DOX+RGD-rmhTNFα (10,000, 50,000, and 250,000 IU/kg, respectively).

To begin further characterizing the *in vivo* antitumor activity of RGD-rmhTNF-α alone and in combination with doxorubicin in the 2 murine tumor models, we first tested the antitumor activity of rmhTNF-α and RGD-rmhTNF-α alone in the absence of chemotherapeutic agents. To compare the antitumor dose-response curves of rmhTNF-α and RGD-rmhTNF-α, we administered various doses (5,000–500,000 IU/kg weight) of each molecule (i.m.) into H22 hepatoma- or S180 sarcoma-bearing mice. Tumor-bearing mice injected with doxorubicin (2 mg/kg weight, i.p.) or saline were used as the positive and negative controls, respectively. As expected, doxorubicin significantly inhibited tumor growth compared to the negative control (*p*<0.05). Although low doses of rmhTNF-α or RGD-rmhTNF-α alone could not inhibit the growth of either tumor type, higher doses (500,000 IU/kg weight) were able to modestly delay tumor growth (*p*>0.05) (Figure 2A and Figure 3A). This observation suggested that administering low doses of rmhTNF-α or RGD-rmhTNF-α alone did not lead to inhibition of tumor growth on their own.

### Low Doses of RGD-rmhTNF-α Enhanced the Therapeutic Effects of Doxorubicin

Even though we observed that low doses of rmhTNF-α or RGD-rmhTNF-α could not inhibit tumor growth when administered alone, we investigated whether a targeted delivery of low doses of RGD-rmhTNF-α into tumor vessels could function to enhance the antitumor activity of chemotherapeutic drugs in the H22 and S180 tumor models. While combination therapy of doxorubicin with low doses of rmhTNF-α did not inhibit H22 or S180 tumor growth (Figure 2A and Figure 3A), the double-agent combination therapy of doxorubicin with RGD-rmhTNF-α at the 50,000 IU/kg dose induced a stronger antitumor effect on H22 tumor-bearing mice compared to that induced by the single doxorubicin agent (Figure 2A). Moreover, doxorubicin synergized with 10,000 IU/kg of RGD-rmhTNF-α to inhibit tumor growth of S180 allografted tumors (Figure 3A). These results indicated that low doses of RGD-rmhTNF-α were sufficient to improve the response of tumors to doxorubicin chemotherapy. These results could also be observed by looking at the gross tumor growth in the representative images from selected experimental groups shown in Figure 2B and Figure 3B.

### RGD-rmhTNF-α Accumulated in Tumor Tissues more than rmhTNF-α

**Figure 4:**
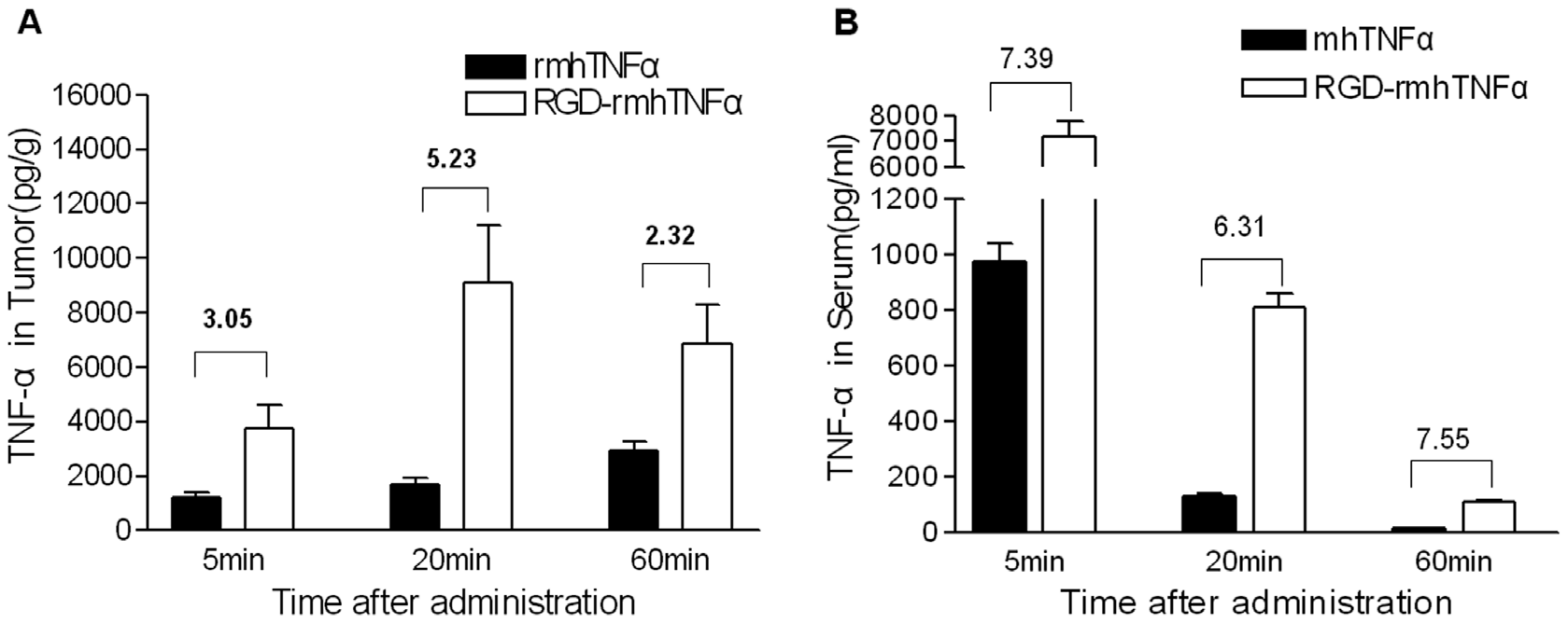
Distribution of RGD-rmhTNF-α and rmhTNF-α in tumors and serum of S180-bearing ICR mice. Equimolar amounts of RGD-rmhTNF-α (5.89 µg/kg weight) and rmhTNF-α (5.52 µg/kg weight) were i.v. injected into the tail vein of S180 hepatoma-bearing ICR mice. TNF-α levels were detected in serum and tumor homogenates by ELISA at 5, 20, and 60 min post-dosing (n = 6 mice/time point. Data are represented as the mean ± SD from 3 experiments. Numbers above bars indicate relative fold changes between the groups.

Since RGD should home to the tumor vasculature, we predicted that the concentration of RGD-rmhTNF-α would be higher than rmhTNF-α within the tumor. To examine whether the intratumoral distribution of RGD-rmhTNF-α differed from rmhTNF-α, equimolar amounts of RGD-rmhTNF-α (5.89 µg/kg) and rmhTNF-α (5.52 µg/kg) were i.v. injected through the tail vein. The concentration of intratumoral RGD-rmhTNF-α was found to be 3.05-, 5.35-, and 2.32-fold higher than that of rmhTNF-α at the 5, 20, and 60 min time points, respectively (Figure 4A). Based on the time-dependent changes in concentration within the serum (Figure 4B), rmhTNF-α rapidly decreased within 1 h, but RGD-rmhTNF-α remained 7.39-, 6.31-, and 7.55-fold higher in the serum than that of rmhTNF-α at the 5, 20, and 60 min time points, respectively. Thus, attaching RGD to rmhTNF-α allows it to be retained longer in the circulation and accumulate more in the targeted tumor tissue than rmhTNF-α alone.

## Discussion

Previously, we showed that rmhTNF-α exhibited lower toxicity and higher antitumor efficacy compared to wild-type TNF-α [10]. Preliminary animal studies showed that rmhTNF-α was a promising and safe therapy to use in the clinic [25]. In addition, neovasculature-targeted rmhTNF-α (RGD-rmhTNF-α)––constructed by fusing the RGD-4C peptide to the N-terminus of rmhTNF-α––not only exhibited higher bioactivity than rmhTNF-α *in vitro* but also bound to ανβ3 integrin, which was known to be significantly up-regulated on endothelial neovascular cells within human tumors [20].

In the present study, we showed that administering RGD-rmhTNF-α in combination with doxorubicin exerted a heightened antitumor response in 2 different tumor models. Several mechanisms could have contributed to this observed synergy. One possible mechanism could be that pre-administering RGD-rmhTNF-α enhanced the permeability of blood vessels within the tumor, allowing for the increased uptake of doxorubicin into the tumors. In general, TNF-α is normally able to rapidly increase endothelial permeability and decrease interstitial fluid pressure in tumors, both of which are believed to overcome significant barriers to drug penetration into tumor tissues [26], [27]. Thus, tumor vessel damage and increased drug penetration are thought to be crucial mechanisms underlying how TNF-α synergizes with chemotherapeutic drugs [4]. Indeed, this mechanism could also help to explain the increased tumor responses to the tumor-vessel–targeted rmhTNF-α in our study, as RGD-rmhTNF-α enhanced the permeability of tumor blood vessels as well as the intratumoral accumulation of doxorubicin. While our data strongly support a role for RGD-rmhTNF-α in increasing vascular permeability, we cannot exclude the possibility that this reagent also facilitates other mechanisms that can enhance doxorubicin levels in the tumors, such as by affecting ABC transporters or other such mechanisms, which should be investigated in future studies.

Another possible mechanism for the enhanced antitumor activity might be the specific targeting of RGD-rmhTNF-α to the tumor. Indeed, administration of a low dose of non-targeted rmhTNF-α combined with doxorubicin did not exhibit a synergistic antitumor effect in the present study. Furthermore, similar to the findings shown here, a previous study showed that low doses of vascular-targeted TNF (NGR-TNF-α) enhanced the penetration of the chemotherapeutic drugs into tumors, improving their efficacy [28]. Also, the concentration of intratumoral RGD-rmhTNF-α was markedly higher than rmhTNF-α as assessed by tumor distribution, further supporting a role for this mechanism. Although RGD-rmhTNF-α has minimal interaction with normal vessels because they do not express αvβ3, it can interact highly with αvβ3 and TNF receptors on the tumor neovasculature, likely leading to selective activation of the endothelial cells and a reduction of drug-penetration barriers within the tumor. However, low-dose rmhTNF-α or RGD-rmhTNF-α could also increase tumor-cell proliferation, as shown in Figs. 2Aand 3A, which could also potentially render the tumors more susceptible to chemotherapy.

Other investigators previously demonstrated increased antitumor activity by combining NGR-TNF-α with melphalan, cisplatin, paclitaxel, or gemcitabine using various schedules and doses in mouse B16F1 melanoma, RMA-T lymphoma, TS/A mammary adenocarcinoma, and WEHI-164 fibrosarcoma [24], [28]. However, to our knowledge, the present study is the first to demonstrate a similar effect using the tumor-homing peptide RGD-4C coupled to the recombinant mutated human TNF-α (RGD-rmhTNF-α). Indeed, RGD-rmhTNF-α treatment increased the vascular permeability of the tumor to Evans Blue or doxorubicin; moreover, decreased tumor burden of both H22 hepatoma and S180 sarcoma tumors in mice was also noted when RGD-rmhTNF-α treatment (10,000–50,000 IU/kg) was followed by doxorubicin (2 mg/kg). One seeming discrepancy in our data was that the 10,000 IU/kg dose that induced the highest antitumor effects on S810 tumors was less than the dose that induced the highest vascular permeability seen in Figure 1A. This was likely due to the different experimental schedules between these experiments, where the readout of vascular permeability was assessed 2 h after a single treatment of RGD-rmhTNF-α, while the antitumor readout of tumor growth was assessed after repeated treatments of RGD-rmhTNF-α over a nearly 2-week period, which might have allowed for other functions of TNF-α to occur that might have diminished the enhanced efficacy of doxorubicin.

The results of our study showed that administration of low-dose rmhTNF-α or RGD-rmhTNF-α alone had no, or only minimal, effect on tumor growth. Previous studies indicate that TNF-α induces not only hyperpermeability of existing blood vessels but also tumor vasculogenesis. This angiogenic function might be due to the ability of TNF-α to cause differentiation of myeloid progenitor cells into endothelial cells within the tumor microenvironment [29]. Therefore, the RGD-rmhTNF-α–mediated increase in the local concentration of chemotherapeutic agents might be the main mechanism underlying the synergistic antitumor effect observed between RGD-rmhTNF-α and doxorubicin.

In conclusion, our results showed that targeted delivery of low-dose RGD-rmhTNF-α to the tumor vasculature not only increased the vascular permeability but also allowed increased entry of doxorubicin into the tumor tissues. Additionally, combined treatment of RGD-rmhTNF-α and doxorubicin synergized and achieved enhanced antitumor efficacy *in vivo*, suggesting that combining RGD-rmhTNF-α with conventional chemotherapeutic agents is a potentially effective therapeutic strategy for cancer. However, future studies are required to further evaluate the antitumor activity and safety of combination-based therapy using RGD-rmhTNF-α with various chemotherapeutic agents in different allografted tumor models. Based on the data presented here, this study provides important insights into designing improved clinical therapies against cancer that combine RGD-rmhTNF-α with chemotherapeutic agents.
